# Therapeutic hypothermia as a neuroprotective strategy in newborns with perinatal asphyxia—case report

**DOI:** 10.3389/fresc.2023.1132779

**Published:** 2023-04-19

**Authors:** Nayara Rodrigues Gomes de Oliveira, Gustavo Gonçalves Teixeira, Kathlen Terezinha Montes Soares Fernandes, Marla Moreira Avelar, Maja Medeiros, Cibelle Kayenne Martins Roberto Formiga

**Affiliations:** ^1^ Department of Physiotherapy, State University of Goiás-UEG, Goiânia, Brazil; ^2^ Department of Medicine, Hospital das Clínicas, Federal University of Goiás-UFG, Goiânia, Brazil

**Keywords:** perinatal asphyxia, hypoxic-ischemic encephalopathy, hypothermia, child development, case report

## Abstract

**Background:**

Perinatal asphyxia is a public health problem and the third major cause of death among children under 5 years.

**Objective:**

Two clinical cases of newborns with perinatal asphyxia submitted to therapeutic hypothermia and the follow-up of their motor development after hospital discharge have been reported.

**Methods:**

This retrospective case report study included two newborns with hypoxic-ischemic encephalopathy due to perinatal asphyxia who received a hypothermia protocol at the neonatal intensive care unit (NICU). The two newborns and their families were followed up at the outpatient clinic and assessed using the Hammersmith Child Neurological Examination, Alberta Child Motor Scale, and Denver Developmental Screening Test-II.

**Results:**

The newborns were submitted to a 72-hour hypothermia protocol. One newborn remained for 13 days in the NICU, while the other remained for 22 days. According to the multidisciplinary team, both cases presented with typical motor development with no cerebral palsy during the follow-up.

**Conclusion:**

Both cases showed positive results and a good prognostic for motor development. Therapeutic hypothermia may be a strategy to prevent neurologic sequelae in newborns with perinatal asphyxia, including cerebral palsy.

## Introduction

Perinatal asphyxia is responsible for 30%–35% of neonatal deaths worldwide, representing 1 million deaths per year (1). Also, it is a public health problem and the third cause of death of children under five years, preceded by prematurity and congenital anomalies (1–3). In 2019, 20% of neonatal deaths were associated with perinatal asphyxia, hypoxia at birth, or meconium aspiration syndrome, which are responsible for four deaths per day of newborns with birth weight >2,500 g in Brazil (4).

Perinatal asphyxia occurs when significant tissue hypoperfusion compromises gas exchange, possibly leading to hypoxemia and progressive hypercapnia with metabolic acidosis (2, 3). Hypoxic-ischemic encephalopathy is the main consequence of perinatal asphyxia and is responsible for the high rates of neonatal mortality and morbidity worldwide, prevalent in about 15 per 1,000 live births (3, 5, 6). Among survivors, 25%–30% develop severe sequelae [e.g., cerebral palsy (CP), functional disability, or cognitive impairment] (3, 7–9).

Therapeutic hypothermia (TH) is a neuroprotective strategy to reduce mortality and disability in children with hypoxic-ischemic encephalopathy due to perinatal asphyxia after 18–24 months of protocol. TH should be initiated within the first 6 hours after birth and consists of reducing the body temperature of newborns (between 33°C and 34°C) for 72 h (7, 9, 10). Hypothermia reduces brain metabolism by approximately 5% per 1°C drop in body temperature, which delays the onset of anoxic cell depolarization (11).

The neuroprotective efficacy and safety of TH depend on controlling comorbid factors (e.g., adequate neonatal resuscitation, early onset of TH, and control of hyperthermia, hypoglycemia, hypercapnia, hyperoxia, and hypocalcemia at the first hours of life). The duration and depth of hypothermia, rewarming, sedoanalgesia, and simultaneous application of other therapies are also determining factors. However, TH requires great intensive care support, which may not be provided in low- and middle-income countries, possibly leading to complications (e.g., mortality, epilepsy, or cognitive impairment) (12, 13).

Several newborns admitted to intensive care units may present with acute neurological injuries due to increased exposure to risk factors. For example, perinatal asphyxia is one of the main risk factors for developing neurological and motor changes in these newborns, which may lead to chronic encephalopathy or CP. Thus, identifying motor and sensory changes related to CP and applying neurocritical care are essential for newborns with perinatal asphyxia (14).

Several instruments and procedures are available to detect childhood disabilities earlier. Clinical guidelines recommend detecting risks of CP before 5 months of corrected age using the following predictive instruments: magnetic resonance imaging (MRI), which indicates brain abnormalities affecting motor tracts; General Movements Assessment (GMA), which analyzes the spontaneous movements of newborns; and Hammersmith Child Neurological Evaluation (HINE), which evaluates tone, posture, reflexes, and functional limitations (15–17).

Studies on the assessment and long-term follow-up of motor development of newborns submitted to a hypothermia protocol are scarce. Thus, this study aimed to report two clinical cases of newborns with perinatal asphyxia submitted to TH and findings from the follow-up of motor development after hospital discharge.

## Methods

This retrospective case report study included two newborns with hypoxic-ischemic encephalopathy due to perinatal asphyxia submitted to a hypothermia protocol at the neonatal intensive care unit (NICU) of a hospital in Goiânia (Goiás, Brazil). A female newborn was born by cesarean section at 39 weeks of gestational age, while a male was born by cesarean section at 37 weeks and 2 days of gestational age. After discharge from the NICU, both newborns were followed up at the high-risk outpatient clinic of the hospital. Data from clinical records were collected, and interviews with caregivers and neurological and developmental assessments of the children were conducted.

Two pediatricians and the physical therapy team assessed the newborns every 3 months during the follow-up. Motor development and possible neurological sequelae were assessed using the HINE, Alberta Child Motor Scale (AIMS), and Denver Developmental Screening Test-II (Denver II). Trained evaluators applied these instruments and followed the recommendations of the guide manuals.

The HINE is a high-sensitivity and internationally validated scale to detect CP and other neurodevelopmental disorders in children between 2 and 24 months of corrected age. Also, it is easy to apply and is considered a high-performance strategy to quantitatively assess the cranial nerve function, posture, movements, tone, and reflexes and reactions. Infants aged between 3 and 6 months are expected to score between 67 and 70 points, and those aged between 9 and 12 months are expected to score 73 points or above. Scores <63 points indicate the need for new investigation, whereas scores ≤26 points indicate neurological sequelae (18, 19).

The AIMS assesses motor development of full-term and preterm children aged between 0 and 18 months using an observational approach related to their behavior in specific antigravity postures to detect possible signs of delayed motor development. Children are assessed in four main postures (i.e., prone, supine, sitting, and standing), and percentiles range between 5 and 90 points. Also, the quantitative assessment allows us to apply early interventions. The assessment and quantitative analysis must be conducted according to the corrected age of the child (20).

The Denver II aims to track the child development to provide comparative parameters in each assessment, allowing the detection of possible delays in the development from 0 to 6 years of corrected age. The test assesses the responses of children in four domains: personal–social, language, gross motor, and fine motor-adaptative (21).

Caregivers were informed about the results after the assessments and received verbal orientations to stimulate exercises for age-related motor development.

The present study followed the Declaration of Helsinki and guidelines for research involving human beings and was approved by the research ethics committee of the hospital. All caregivers signed the informed consent form.

## Results

### Clinical case 1

The first case is a newborn, female, born by cesarean section at 39 weeks of gestational age, weighing 3,055 g, and 46.5 cm in length. The mother was diagnosed with panic disorder, used long-term sertraline, and had prelabor rupture of membranes. The newborn presented Apgar scores of 7 and 8 at the first and fifth minutes (respectively), requiring positive pressure ventilation (PPV). She evolved with respiratory distress and groaning; thus, 30% oxygen was administered using a hood for 1 hour, followed by 3 hours of continuous positive airway pressure (CPAP) with a 40% fraction of inspired oxygen due to lack of improvement. After four hours of birth, the newborn presented with worsened respiratory distress (70% of oxygen saturation) and cyanosis in the extremities, requiring intubation. During intubation, she had a seizure episode with hyperextension of the upper limbs and internal rotation of the wrists. The newborn received an anticonvulsant and did not have new episodes.

The newborn presented with respiratory disorder, transient tachypnea, and neurological disorders (i.e., perinatal asphyxia and hypoxic-ischemic encephalopathy). Tests indicated perinatal asphyxia, and the TH protocol was applied. The incubator was turned off for 72 h until the body temperature reached between 33°C and 34°C, which was monitored every 30 min. She was diagnosed with late neonatal sepsis in the NICU, requiring antibiotics for seven days. Transfontanelle ultrasonography indicated a reduction of the sulci and diffuse hyperechogenicity. After 7 days, the MRI of the skull indicated sequelae of cerebral palsy due to the severe perinatal hypoxic-ischemic event.

The newborn remained in the NICU for 13 weeks, and 11 days in the ward. She was discharged with a drug prescription and indication for breastfeeding and milk formula. At discharge, neurological examination indicated mild generalized hypotonia and symmetrical primitive reflexes (i.e., rooting, palmar and plantar grasp, Moro, and tonic neck reflexes).

The follow-up of the child started at the pediatric clinic in November 2021 at 18 months old. The child presented with good general condition and no signs of neurological sequelae. The two assessments (at 18 and 27 months of chronological age) using the Denver II indicated typical development and risk of delay in the language domain because she failed in the items “points to six body parts” and “speech half understandable”. She presented with typical age-related development in the other domains of the test. Caregivers were instructed to enrich the home environment to stimulate speech by reading, music, talking while playing, using sonorous toys, and encouraging the child to participate in games and walks with other children.

### Clinical case 2

Case 2 is a newborn, male, born by cesarean section at 37 weeks and 2 days of gestational age, weighing 2,920 g and 47 cm in length, with Apgar scores of 2, 3, 4, and 7 in the 1st, 5th, 10th, and 15th minutes, respectively. The mother was 31 years old and had five pregnancies and one abortion. During the prenatal period, she developed placental abruption, urinary tract infection, arterial hypertension, gestational diabetes mellitus, and a history of chronic asthma.

The mother had a difficult delivery, resulting in perinatal asphyxia in the newborn, who required two cycles of PPV. The newborn evolved with intense respiratory distress and received CPAP, which was not well tolerated. Thus, he was intubated and diagnosed with chronic hypoxic-ischemic encephalopathy. Then, arterial and venous umbilical catheters were implanted. After analyzing the cord blood gas and scoring 4 points on the Sarnat score (i.e., moderate encephalopathy), he was eligible for the TH protocol, which was performed for 72 h with 33°C of body temperature.

After the delivery, the newborn was transferred to the NICU, where he was hypoactive and hyporeactive to handling, submitted to PPV, and monitored with pulse oximetry. The newborn received phototherapy for 3 days due to late neonatal jaundice, and his hyperglycemia was controlled with insulin therapy. He also presented with seizures, which were controlled after adjusting the anticonvulsant.

The MRI of the skull demonstrated asymmetry of the occipital horns of the lateral ventricles (i.e., the left was larger than the right) and a possible frontoparietal subgaleal hematoma. The echocardiogram indicated situs solitus, collapsible inferior vena cava, septal hypertrophy, subjective assessment of adequate contractility, and tricuspid valve regurgitation. Also, the right lung presented a mild perihilar infiltrate in the chest radiography. The newborn was discharged after 22 days of hospitalization in good general condition, eupneic, with normal color, hydrated, acyanotic, anicteric, afebrile, active, and reactive, with drug prescription, breastfeeding, and milk formula.

The follow-up of the child started at the pediatric clinic in March 2022, at 6 months and 18 days of chronological age. The assessments of motor development were performed every 3 months using the AIMS, HINE, and Denver II. The three tests were applied in the first assessment (at 6 months and 18 days of chronological age), and the child scored 64 points in the HINE: 15 in the cranial nerve function, 16 in posture, 6 in movements, 18 in tone, and 9 in reflexes and reactions. He scored 19 points (<5 points of percentile) in the AIMS and did not present delays in the Denver II.

In his second assessment (at 9 months and 17 days of chronological age), only the AIMS and Denver II were applied. “Although the child improved the achievement of motor skills in the AIMS (increasing the score to 32 points; <5 points of percentile), the test indicated delay in motor development.” In the Denver II, he only presented delay in the language domain for the items “combines syllables” and “uses jargon.” Caregivers were instructed to enrich the home environment to stimulate the child.

In the last follow-up (at 11 months and 22 days of chronological age), the child presented with good general condition and no signs of neurological sequelae. In the AIMS, he improved the achievement of motor skills, scoring 52 points 50 points of percentile) and showed typical behaviors for his age in the four domains of the Denver II.

**Figure 1:**
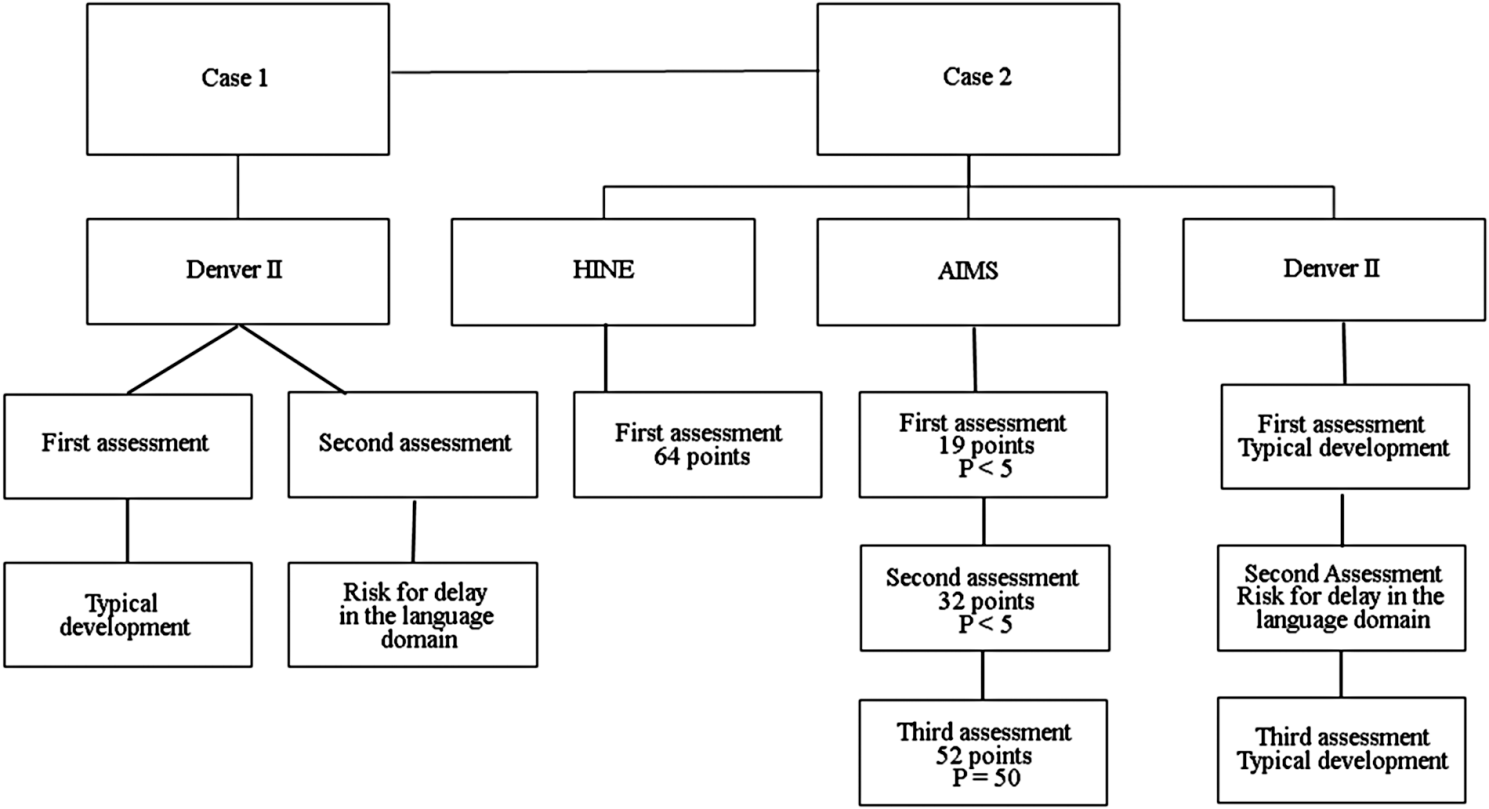
Instruments to assess the two cases. P, percentile.

Results of the TH protocol for both case reports are shown in Table 1, and results of the assessments performed in the follow-up are shown in Figure 1.

**Table 1:** Results of the TH protocol for both case.

Variable	Case 1	Case 2
Gestational age	39 weeks	37 weeks and 2 days
Type of delivery	Cesarean	Cesarean
Prenatal complications	None	Placental abruption, urinary tract infection, arterial hypertension, gestational diabetes mellitus, and history of chronic asthma
Complications during labor	None	Decompensated gestational diabetes mellitus and difficult delivery, requiring the use of forceps
Birth weight (g)	3,055	2,920
Apgar score (1st, 5th, 10th, and 15th min)	7 and 8	2, 3, 4, and 7
Ventilatory support	Hood (30% oxygen), CPAP, MV, NO_2_	MV, CPAP
Duration of hypothermia (h)	72	72
Cooling temperature (rectal temperature)	33–34°C	33°C

CPAP, continuous positive airway pressure; MV, mechanical ventilation; NO_2_, nasal oxygen.

## Discussion

TH is a promising strategy for several consequences of perinatal asphyxia in newborns with gestational age lower or greater than 36 weeks. Also, the protocol has been used as a neuroprotection strategy to prevent secondary changes in the neurological status of newborns (22). The main eligibility criteria for the use of TH protocol are perinatal asphyxia related to the continuous need for resuscitation (with or without ventilatory support) in the first minutes after birth and Apgar score ≤5 (22). In the present study, both newborns met the eligibility criteria for the protocol (gestational age >36 weeks and diagnosed with hypoxic-ischemic encephalopathy). In case 1, the newborn showed signs of respiratory distress and groaning, requiring one cycle of PPV. After 4 h, the newborn showed signs of seizures, the exams evidenced perinatal asphyxia, and TH was applied 6 h after delivery. In case 2, the newborn had perinatal asphyxia, requiring two cycles of PPV. Also, cord blood gas and the Sarnat test were analyzed, indicating moderate encephalopathy.

In randomized clinical trials including newborns with hypoxic-ischemic encephalopathy and gestational age >36 weeks, they were submitted to a whole-body cooling (33.5°C) at the beginning of the protocol, remaining at this temperature for 72–96 h. After 96 h, the newborns were rewarmed by increasing 0.5°C per hour (8, 9). In the present study, the newborn described in case 1 was kept at a temperature between 33 and 34°C, while the other newborn was kept at a minimum temperature of 33°C (both for 72 h) maintaining a similar TH protocol to literature.

Both newborns had positive results in the management of CP. The newborn in case 1 was hospitalized for 13 weeks and 11 days, while the other was hospitalized for 22 days; both were discharged without ventilatory support. These results corroborated a study assessing the effects of TH in 216 newborns with severe or moderate hypoxic-ischemic encephalopathy and gestational age between 39 and 40 weeks. They showed reduced mortality, duration of hospitalization (reduced by 5.2 days per newborn), lesion size, and anticonvulsant prescription, evidencing the TH in neurocritical care (22).

Longitudinal follow-ups of both newborns are needed to detect the long-term effects of TH and evaluate their development, considering the high risk of these cases. Also, the follow-up is an excellent strategy to identify possible delays in motor development in an interdisciplinary approach and assist the early diagnosis using specific instruments when needed (23). In this study, the follow-up outpatient clinic comprised pediatricians and physical therapists who monitor high-risk newborns until 5 years old.

Studies suggested three main instruments for the early detection of CP: MRI (86%–89% sensitivity), GMA (98% sensitivity), and HINE (90% sensitivity) (15–17). In case 2, HINE was applied at 6 months of age and the newborn scored 64 points, suggesting a neuromotor delay according to the expected score for the age. However, he presented good motor development for the age and few findings compatible with CP after the TH protocol, considering the sensitivity and specificity of the scale.

Although newborns with severe perinatal asphyxia are expected to show all signs of disability and poor markers related to motor development due to CP, neuroprotection strategies provide a positive prognosis in the development. Although newborns with severe perinatal asphyxia are expected to show all signs of disability and poor markers related to motor development due to CP, neuroprotection strategies may provide positive prognosis in the development. In this sense, a study assessed newborns with perinatal asphyxia divided into normothermia and hypothermia groups. The newborns who survived were followed up at 3, 6, 9, 12, and 18 months of corrected age using the Developmental Assessment Scale for Indian Childs. The authors reported improved motor development in children submitted to hypothermia compared with children in the normothermia group at 18 months of life, corroborating our results. These results may explain the positive outcomes of development in our study since both newborns improved their behaviors throughout the assessments (14). The authors reported improved motor development in children submitted to hypothermia compared with children in the normothermia group at 18 months old, corroborating our results (14). These results may explain the positive outcomes of development in our study since both newborns improved their behaviors throughout the assessments.

This study presented some limitations, such as the nonstandardized application of all instruments in the two clinical cases. Also, the child in case 2 was admitted to the outpatient clinic at more than 6 months old, whereas the other newborn in case 1 was 1 year and 6 months old, hindering the early assessment of both. Another limiting factor was the lack of MRI exam during the follow-up, which was performed only in the NICU.

## Conclusion

The cases described two newborns diagnosed with hypoxic-ischemic encephalopathy due to perinatal asphyxia who received TH protocol for 72 h with strict body temperature monitoring. They were followed up at the outpatient clinic by a multidisciplinary team, and the assessment of motor development indicated that both newborns had typical motor development in the assessed domains and no clinical neurological sequelae. Results suggested that neonatal hypothermia can be used as a neuroprotective strategy in newborns with perinatal asphyxia, minimizing and preventing neurological sequelae, such as CP.

## Data Availability

The raw data supporting the conclusions of this article will be made available by the authors, without undue reservation.
